# The anti-fatigue and sleep-aiding effects vary significantly among different recipes containing *Ganoderma lucidum* extracts

**DOI:** 10.1016/j.heliyon.2024.e30907

**Published:** 2024-05-08

**Authors:** Kexin Li, Wenzhen Liu, Changhui Wu, Le Wang, Yunmei Huang, Ye Li, Huimin Zheng, Yanyu Shang, Lei Zhang, Zhuo Chen

**Affiliations:** a State Key Laboratory of Structural Chemistry, Fujian Institute of Research on the Structure of Matter, Chinese Academy of Sciences, Fujian Academy, University of Chinese Academy of Sciences, Fuzhou, Fujian, 350108, China; b Fujian Xianzhilou Biological Science and Technology Co. Ltd., Fuzhou, Fujian, 350108, China; c Academy of Integrative Medicine, Fujian University of Traditional Chinese Medicine, Fuzhou, 350122, China; d College of Life Sciences, Fujian Agriculture and Forestry University Fuzhou, Fujian, 350002, China; e College of Biological Science and Engineering, Fuzhou University, Fuzhou, Fujian, 350108, China; f University of Chinese Academy of Sciences, Beijing, 100049, China

**Keywords:** *Ganoderma lucidum* extract, anti-Fatigue activity, Sleep-aiding activity, Glycogen levels, Serum lactic acid, Serum urea nitrogen, Malondialdehyde

## Abstract

**Aims:**

This study aims to delve into the anti-fatigue and sleep-aiding effects of various formulations containing *Ganoderma lucidum* extracts.

**Materials and methods:**

PGB [incorporating *Ganoderma lucidum* extract (GE), broken *Ganoderma lucidum* spore powder (GB) and *Paecilomyces hepiali* mycelium (PH)] and GBS [composed of GE, GB, and *Ganoderma sinense* powder (GS)] were chosen as representative recipes for this study. Mice were treated with these recipes or key components of *Ganoderma lucidum* for 14 consecutive days. Subsequently, a weight-bearing swimming experiment was conducted to assess the mice's exhaustion time and evaluate the anti-fatigue properties of the recipes. Sleep-aiding effects were analyzed by measuring the sleep latency and duration. Furthermore, levels of blood lactic acid, serum urea nitrogen, hepatic glycogen, muscle glycogen, and malondialdehyde (MDA) were measured in the livers and muscles.

**Key findings:**

The anti-fatigue abilities of the tested mice were significantly improved after treatment with PGB and their sleep quality improved as well with GBS treatment. PGB treatment for 14 days could significantly prolong the exhaustion time in weight-bearing swimming (from 10.1 ± 0.5 min to 15.2 ± 1.3 min). Meanwhile, glycogen levels in the livers and muscles were significantly increased, while the levels of serum lactic acid, serum urea nitrogen, and MDA in the livers and muscles were significantly decreased. In contrast, mice treated with GBS for 14 days experienced significant improvements in sleep quality, with shortened sleep latency (from 6.8 ± 0.7 min to 4.2 ± 0.4 min), extended sleep duration (from 88.3 ± 1.4 min to 152.5 ± 9.3 min), and decreased muscle MDA levels. These results indicated that *Ganoderma lucidum* extracts can be used for anti-fatigue and or aid in sleeping, depending on how they are prepared and administered.

**Significance:**

This study provides experimental evidence and theoretical basis for the development of *Ganoderma lucidum* recipes that are specifically designed to help with anti-fatigue and sleep.

## 1 Introduction

In today's fast-paced modern life, work-related stress, irregular eating habits and sleep patterns can lead to fatigue and insomnia. It is reported that these conditions affect approximately one-third of the global population [[1], [2], [3]].

Fatigue is a highly intricate physiological and biochemical process. According to existing beliefs, the primary mechanisms underlying exertional fatigue involve energy depletion, the accumulation of metabolites, and oxygen-free radical-induced lipid peroxidation [[4], [5], [6]]. Timed weight-bearing swimming can be an indicator that reflects prolonged or intense exercise, which directly correlates with physical endurance, and are crucial metric in assessing the degree of fatigue [7,8].

The mechanisms of sleep are sophisticated and involve numerous factors. A study has shown that relaxation or fatigue upon waking up is a crucial subjective factor for individuals to assess their sleep quality [9]. Objective criteria, such as total sleep time (TST), sleep latency (SOL), sleep maintenance, total wakefulness time (TWT), and sleep efficiency (SE), provide quantitative measures for evaluating sleep quality. Additionally, sleep disorders like spontaneous wakefulness or respiratory pauses are also considered [10]. While these criteria may appear superficial, they reflect deeper metabolic processes that ultimately impact apparent sleep parameters like sleep time and latency. According to previous research [11,12], a decrease in glycogen levels can lead to increased sleep stress. Similarly, an increase in malondialdehyde (MDA), a marker of lipid peroxidation, has been associated with heightened sleep stress [13]. Kidney disease patients, who often have elevated blood urea nitrogen levels, are prone to sleep disorders [14]. Earlier reports have also linked serum lactate levels to sleep quality [15,16]. However, these relationships are complex and not fully understood. One specific aspect that has garnered attention is the role of reactive oxygen species (ROS). The accumulation of ROS in the body can lead to oxidative stress and increase sleep stress [17]. In this study, we focused on investigating how two specific formulas comprised of *Ganoderma lucidum* extracts, affect metabolic changes after consumption and their potential anti-fatigue and sleep-aiding effects. Based on extensive previous research, we selected MDA as a key indicator for its potential role in affecting sleep through metabolic alteration. Additionally, sleep latency, defined as the duration it takes to transition from a conscious state to a sleep state, is an objective measure of sleep quality [18]. A sleep latency of 16-30 min is generally considered optimal for achieving good sleep quality. Conversely, a latency exceeding 1 h often indicates poor sleep quality [19]. Sleep duration, on the other hand, refers to the total amount of time spent sleeping, excluding any periods of waking during that time [18,20]. For adults, it is recommended to have at least 7 h of continuous sleep to maintain good health [21]. However, it is estimated that approximately two-thirds of adults worldwide do not get sufficient sleep time [22]. Insomnia, or the inability to fall asleep or maintain sleep, not only disrupts normal work and daily activities but can also lead to endocrine disorders, which can have serious health implications [23,24]. Chronic sleep deprivation and the inability to combat fatigue can lead to multiple health risks, including depression, cancers, and various other diseases [25,26]. Therefore, ensuring adequate sleep time and quality is crucial for maintaining overall health and well-being.

Anti-fatigue and sleep are crucial self-repair functions of the body, involving intricate energy and material conversions. Several physiological performance indicators are intimately linked to feelings of fatigue or insomnia. Glycogen reserve, for instance, serves as a vital energy source for human activity and is intricately connected to exercise capacity [27]. During exercise, the depletion of muscle glycogen is a key contributor to the sensation of fatigue [28]. Likewise, hepatic glycogen depletion can lead to a decrease in blood glucose levels, ultimately causing energy deficiency and fatigue within the central nervous system [29]. The consumption of energetic substances during exercise is often accompanied by the generation of metabolites [30]. Among these, sugars undergo anaerobic glycolysis to produce lactate, which can accumulate and cause muscle soreness, impairing exercise capacity and leading to inadequate energy supply [31]. When the body's sugar or fat reserves are insufficient to meet the energy demands of exercise, the catabolism of proteins and amino acids is intensified. This process results in an elevation of serum urea nitrogen levels [32], which can further promote muscular fatigue and reduce exercise endurance [33]. Lack of sleep also has a deleterious effect on metabolic function, leading to an increase in serum urea nitrogen content [34]. Furthermore, both high-intensity exercise and insomnia can trigger oxidative stress, damaging organs, cells and macromolecular substances [[35], [36], [37]]. The accumulation of oxygen free radicals attacks unsaturated fatty acids, damaging cell membranes and leading to the production of MDA [38]. This damage to cell membranes hinders the release of adenosine triphosphate, resulting in energy deficiency, the generation of fatigue, and the exacerbation of insomnia [39].

Currently, many individuals resort to energy drinks (ED) such as caffeine- and sugar-enriched beverages to combat fatigue [40]. However, these drinks come with their own set of side effects. Long-term consumption of EDs can have severe health consequences, particularly for children and adolescents [41]. Reported adverse effects include tachycardia [41], headache [42], insomnia [43], drowsiness, and even anger [44]. Treating insomnia often involves the use of sedative hypnotics, primarily benzodiazepines (like estazolam, triazolam and flurazepam) and non-benzodiazepines (such as zolpidem, zaleplon and eszopiclone). Nevertheless, these medications are associated with a range of side effects, including headache, dizziness, rebound insomnia, and the potential for abuse and dependency [3,45]. Supplements aimed at improving sleep often contain melatonin, a hormone naturally produced by the central nervous system. However, melatonin's short elimination half-life restricts its effectiveness as a hypnotic agent [46]. Additionally, the use of animal hormones in these supplements may pose safety concerns and carry the risk of developing dependency in humans [47,48].

At present, there is a scarcity of drugs that possess both anti-fatigue and hypnotic properties. Natural medicines, particularly fungal extracts, have garnered increasing attention due to their low toxicity and minimal side effects [49,50]. Various fungi have demonstrated diverse pharmacological benefits. For instance, *Gymnadenia conopsea* exhibits a broad range of pharmacological effects, including nourishing, anti-fatigue, antioxidant, antiviral, sedative and hypnotic activities [51]. Meanwhile, *Hericium* and *Gastrodia* have rich biological functions such as neuroprotection, anti-fatigue and anti-tumor activities [52,53]. Furthermore, *Poria cocos* and *Lycium barbarum* are known for their anti-tumor, anti-inflammatory, antioxidant, sedative and hypnotic effect [54,55].

*Ganoderma lucidum*, a fungus belonging to the genus *Ganoderma* within the order Basidiomycetes, holds a revered position in Chinese culture as one of the “four cactus plants”. This fungus has long been utilized as a medicinal and edible fungus due to its various bioactive components [56,57]. Its chemical composition includes polysaccharides, triterpenes, nucleosides and other active substances [58,59], granting it sedative, hypnotic, anti-inflammatory and anti-diabetes properties [60,61]. It has traditionally been employed in the treatment of restlessness, insomnia and other health issues [62]. Specifically, polysaccharides extracted from *Ganoderma lucidum* are known to exhibit antitumor, antioxidant, immunomodulatory, and hypoglycemic effects [63,64]. On the other hand, triterpenes isolated from this fungus possess anti-inflammatory, hepatoprotective, and immune-regulating activities [65,66]. In the Chinese Pharmacopoeia, both *Ganoderma sinense* and *Ganoderma lucidum* are listed under the genus *Ganoderma* [62]. Notably, *Ganoderma sinense* exhibits similar pharmacological activities to *Ganoderma lucidum* and is commonly used for liver protection, anti-inflammatory treatments, analgesia, and as an antidote for toxic mushroom poisoning [67]. Various recipes incorporating *Ganoderma lucidum*, with different combinations of ingredients, have demonstrated diverse healthcare benefits. These recipes have been proven to possess anti-tumor [68], anti-oxidation [69], and immune-regulating properties [70]. However, there is still a paucity of research exploring the precise mechanism underlying its anti-fatigue and sleep-aiding effects. According to established research, polysaccharides extracted from *Ganoderma lucidum* effectively reduce post-exercise fatigue by decreasing levels of serum urea nitrogen, serum lactic acid and MDA [71]. Additionally, extracts of *Ganoderma lucidum* have been proven to potentiate the sleep-inducing effects of pentobarbital through the GABA mechanism [72]. *Paecilomyces hepiali* mycelium (PH), known as cordyceps fungus, is derived from the esteemed *Cordyceps sinensis* [73]. This fungus holds a preeminent reputation for its bioactive alkaloids, cyclic dipeptides, steroids, and other bioactive substances [74], all of which contribute to its remarkable medicinal properties.

In this study, we selected two *Ganoderma lucidum*-based recipes, namely PGB and GBS to explore the distinct effects of *Ganoderma lucidum* extract in different formulations and to investigate the underlying mechanisms of their anti-fatigue and sleep-aiding properties. PGB, a formulation primarily composed of *Ganoderma lucidum* extract (GE), broken *Ganoderma lucidum* spore powder (GB), and *Paecilomyces hepiali* mycelium (PH), incorporates 25% of GE, 25% of GB and 50% of PH. Notably, PGB also contains 0.08% of total polysaccharides. These substances are credited with pharmacological and clinical effects such as anti-fatigue, anti-oxidation, and anti-bacterial activities [[75], [76], [77]]. GBS, another formulation, comprises GE, GB and *Ganoderma sinense* powder (GS). Its active substances include 12.5% of total polysaccharides and 1% of Ganoderma lucidum triterpenes. By studying these two recipes, we aim to provide an experimental basis for the application of *Ganoderma lucidum* extract in anti-fatigue and sleep-aiding formulations.

## 2 Materials

### 2.1 Materials and reagents

PGB and GBS are two recipes that incorporate various ingredients with specific percentages and active substances (Table 1). PGB comprises 25% of GE, 25% of GB and 50% of PH. PGB also contains 0.08% of total polysaccharides, a type of carbohydrate that is known for its beneficial effects in various biological systems. On the other hand, GBS, another recipe, contains 50% of GE, 25% of GB, and 25% of GS. Its active substances include 12.5% of total polysaccharides and 1% of *Ganoderma lucidum* triterpenes. Triterpenes are a class of compounds known for their biological activities and potential health benefits. Both PGB and GBS recipes were provided by Fujian Xianzhilou Biological Science and Technology Co. Ltd., a company located in Fujian, China.

**Table 1:** Ingredients of different *Ganoderma lucidum* recipes.

Recipe	Dose
PGB	253.0 mg/kg (low dose) containing PH (126.5 mg/kg), GE (63.25 mg/kg), GB (63.25 mg/kg)
	506.0 mg/kg (high dose) containing PH (253.0 mg/kg), GE (126.5 mg/kg), GB (126.5 mg/kg)
GBS	253.0 mg/kg (low dose) containing GE (126.5 mg/kg), GB (63.25 mg/kg), GS (63.25 mg/kg)
	506.0 mg/kg (high dose) containing GE (253.0 mg/kg), GB (126.5 mg/kg), GS (126.5 mg/kg)

### 2.2 Experimental animals and instruments

SPF (specific pathogen-free) ICR mice, aged 5-6 weeks and weighing 22.2 ± 2.2 g, were purchased from Shanghai Slack Laboratory Animal Co., Ltd., holding a production license number of SCXK (Shanghai) 2017-0005 and a qualification certification certificate number of 2015000547947. The mice were maintained in a controlled environment with a constant temperature of 25°C and a regular 12/12-h light-dark cycle, with free access to food and water. The light phase was set from 08:00 to 20:00 throughout the duration of the experiment. All experimental procedures adhered strictly to the Guidelines for Care and Use of Laboratory Animals, which had been approved by the Animal Ethics Committee of Fujian University of Traditional Chinese Medicine. The primary equipment utilized in the experiment comprises an electronic balance from Sardolis Scientific Instruments (Beijing) Co., Ltd., and a high-speed centrifuge manufactured by Eppendorf China Ltd., and a Synergy 4 multimode microplate reader provided by Shanghai BioTek Biological Technology Co., Ltd.

## 3 Methods

### 3.1 Group and gavage administration

The mice were randomly distributed into ten groups, with eight mice per group. Four groups were administered two distinct *Ganoderma lucidum* recipes (PGB or GBS) in either a low dose of 253 mg/kg body weight or a high dose of 506 mg/kg body weight, consecutively for 14 days. Another five groups were treated with the individual components (PH, GB, GE-PGB, GE-GBS or GS) at a high dose of 506 mg/kg body weight for 14 consecutive days. Meanwhile, the control group mice received 0.9% saline (0.20 mL/10 g) for the same duration. All mice were weighed daily throughout the experiment.

### 3.2 Preparation of GE

The preparation of GE followed a meticulous process. Firstly, mature *Ganoderma lucidum* was harvested, thoroughly cleaned and air-dried. Subsequently, it underwent further drying at 65°C, resulting in a soft, crushable material. For the initial alcohol extraction, a concentrated mixture of ethanol (90-95%) was employed, with a weight 8 to 10 times that of the raw *Ganoderma lucidum*. This extraction was conducted in a slightly boiling state, specifically referring to the initial boiling point of the alcohol when fine bubbles began to roll across the liquid surface. The process lasted for 1.5-3 h, and the resulting mixture was filtered through a 200-mesh silk cloth. The filtrate was then set aside for later use.

In the second ethanol extraction phase, a slightly diluted ethanol mixture of 80-90% was utilized, with a weight of 6-8 times that of the raw *Ganoderma lucidum*. This extraction also took place in a slightly boiling state, lasting for 1.5-2 h. The mixture was again filtered through a 200-mesh silk cloth and combined with the filtrate obtained from the first extraction.

Following the ethanol extractions, a water-based extraction was carried out. Here, water was added in an amount 10 to 12 times the weight of *Ganoderma lucidum* and extracted for 2-3 h at 90-95°C. This mixture was then filtered using a 200-mesh silk cloth and combined with the previously obtained ethanol filtrates. The combined filtrate was concentrated to 10-15% of its solid content. Finally, spray drying was performed, with an inlet temperature of 180 ± 5°C and an outlet temperature of 80 ± 5°C.

### 3.3 Preparation of GB

The preparation of GB followed a distinct process. Initially, the collected mature *Ganoderma lucidum* spore powder was sieved through a 200-mesh sieve to eliminate impurities. Subsequently, the spore powder underwent drying using a gradient heating method. The temperature was gradually increased, starting from room temperature and escalating by 5°C each time, followed by increments of 5–10°C. The drying process continued until the moisture content of the powder reached ≤6%, maintaining a temperature of 65°C. During this drying period, the tray containing the powder was periodically flipped, and an extrusion wall-breaking machine was utilized to gently break the spore wall at a temperature below 10°C. Finally, the *Ganoderma lucidum* spore powder, with a wall-breaking rate of ≥99%, was collected after passing through a 120-mesh sieve.

### 3.4 Preparation of GS

For the preparation of GS, mature purple mushrooms were first gathered, thoroughly cleaned and air-dried. Subsequently, they were further dried at 65°C to ensure complete dehydration. The mushrooms were then subjected to a multi-step crushing process, beginning with a hammer crusher to break down the larger pieces. This was followed by a universal crusher, which further reduced the particles to a finer powder. Finally, a vibration mill was used to achieve ultra-fine crushing, resulting in a powder of exceptional fineness.

After the crushing process, the ultrafine purple powder was sifted through a 200-mesh sieve to ensure a uniform and refined texture. The collected powder, now ready for the final stage, was baked at 65°C until the moisture content reached ≤6%. This meticulous preparation ensured the high quality and purity of the GS, ready for its intended use.

### 3.5 Preparation of PH

To prepare PH, the strain of *Paecilomyces hepiali*, which was kindly provided and identified by the Strain Preservation Center of the Institute of Microbiology, Chinese Academy of Sciences, underwent a meticulous fermentation process. This involved both primary and secondary fermentation stages, each critical in ensuring the optimal growth and activity of the strain.

After fermentation, the broth containing the active components was extracted and separated using a programmed diaphragm filter press. This ensured the efficient removal of solids while retaining the desired bioactive compounds. The extracted liquid was then dried using a spiral belt vacuum dryer, which gently removed excess moisture while preserving the integrity of the bioactive materials. This drying process was followed by a fluidized bed dryer, operated at a temperature of 75 ± 5°C, to achieve a consistent and uniform drying effect. Once dried, the material was crushed into a fine powder and screened to remove any particles that did not meet the desired size specifications. The screened powder was then mixed to ensure homogeneity before being packaged, ready for further use or distribution.

### 3.6 Preparation of PGB and GBS

To prepare PGB, the three key raw materials of PH, GE and GB were mixed in specific weight ratios: PH accounted for 55% of the total, GE made up 25%, and GB comprised the remaining 25%. This meticulous blending of ingredients ensures the desired balance and effectiveness of the final product. After the materials were accurately measured and mixed, they were poured into a U-shaped mixing machine. This type of mixer is chosen for its ability to thoroughly blend materials of different densities and particle sizes, ensuring a uniform mixture. The mixing process lasted for 30 min, allowing the materials to fully integrate and achieve a homogenous consistency. Once the mixing was complete, the resulting mixture, now known as PGB, was ready for further processing or packaging, depending on its intended use.

Similarly, for the preparation of GBS, a different combination of raw materials was used: GE accounted for 55%, GB made up 25%, and GS comprised the remaining 25%. These materials were also mixed in a U-shaped mixer for 30 min to ensure a thorough and uniform blend. The resulting GBS mixture was then ready for further handling or use as required.

### 3.7 Determination of triterpenoids

To determine the triterpenoid content in the sample, a precise amount of approximately 0.2 g of the sample was weighed and placed in a 250-mL conical flask. The addition of 80 mL of ethyl acetate was followed by ultrasonic shaking for 30 min to extract the triterpenoids effectively. The resulting mixture was then filtered to remove any insoluble particles. The filtered extract was transferred to a 100-mL volumetric flask and diluted with ethyl acetate until the liquid reached the scale line, ensuring accurate measurement. From this diluted solution, 1 mL of filtrate was precisely suctioned into a 10-mL test tube. The extract in the test tube was then evaporated to dryness on a 100°C water bath to remove any remaining solvent. Once dry, 0.4 mL of 5% vanillin glacial acetic acid and 1.0 mL of perchloric acid were added to the residue. The mixture was shaken evenly to ensure a thorough mixing of the reagents with the extracted triterpenoids. The mixture was then heated in a 60°C water bath for 15 min to facilitate the reaction between the vanillin and the triterpenoids. After heating, the mixture was immediately transferred to an ice water bath to cool for 3 min, quickly arresting the reaction. Subsequently, 5.0 mL of glacial acetic acid was added to the cooled mixture, and it was shaken evenly. The solution was then allowed to stand at room temperature for 15 min to ensure the stability of the color developed. Finally, the absorbance of the sample solution was measured with a spectrophotometer at a wavelength of 548.1 nm, referencing a standard curve to determine the concentration of triterpenoids. Using this information, the total triterpene content in the original sample, expressed as mg/100 g, was calculated according to the following formula:

$$X_{A}=\frac{\left({W_{A}}_{1}-W_{A2}\right)}{M_{A}\times \frac{{V_{A}}_{2}}{{V_{A}}_{1}}}\times 100$$

where WA1 is the mass (mg) of total triterpenoids in the sample determination solution, obtained by checking the absorbance against the standard curve; WA2 is the mass (mg) of total triterpenoids in the sample blank solution, also obtained from the standard curve. This accounts for any background or interference that may be present in the reagents or the procedure itself; MA is the mass (g) of the original sample taken for the determination; VA1 is the total volume (mL) of the sample solution prepared for determination. In this case, it would be the volume of the diluted solution in the 100-mL volumetric flask, which is 100 mL; VA2 is the volume (mL) of the sample determination solution taken for colorimetry. In this case, it is the 1 mL of filtrate that was evaporated and later used for the color reaction. By following this formula, an accurate and reliable measurement of the total triterpenoid content in the sample can be obtained.

### 3.8 Determination of polysaccharides

To determine the polysaccharide content, the following procedure was executed: Initially, 1.0 g of the sample was placed in a round-bottomed flask and approximately 80 mL of water was added. This mixture was then heated and refluxed in an electric heating jacket for a duration of 2 h. Subsequently, it was cooled to room temperature and filtered, with the filter residue being washed using 10 mL of water. The washing solution was subsequently incorporated into the filtrate, which was then transferred to a 100-mL volumetric flask and diluted to the desired scale.

Next, 5.0 mL of the filtrate was precisely extracted and placed in a 50-mL centrifuge tube. To this, 20 mL of anhydrous ethanol was added and mixed thoroughly. The resulting mixture was centrifuged at 3000 x*g* for 5 min, and the supernatant was discarded. The residue was then washed with several milliliters of an 80% ethanol solution, and the supernatant was discarded again after centrifugation. This washing and centrifugation process was repeated 3 to 4 times. Subsequently, the residue was dissolved in water and diluted to 100 mL. After thorough mixing, 2.0 mL of the sample measuring solution was accurately aspirated into a 25-mL colorimetric tube. To this, 1.0 mL of a 50 g/L phenol solution was added and mixed using a rotary mixer. Carefully, 10.0 mL of concentrated sulfuric acid was added, and the mixture was mixed thoroughly on the rotary mixer before being boiled in a boiling water bath for 2 min. Once cooled to room temperature, the absorbance value was measured using a spectrophotometer at a wavelength of 485 nm. By referencing the standard curve, the quality of dextran was determined, and the content of crude polysaccharides in the sample was subsequently calculated. Additionally, a sample blank test was conducted to ensure the accuracy of the measurements.

### 3.9 Weight-bearing swimming time of mice

The exhaustive swimming test was conducted according to a standard protocol [78]. Briefly, following the administration of the final dose, the mice were securely tethered to a lead block (weighing approximately 5% of their body weight) and gently placed into a swimming tank filled with water to a depth of 30 cm and maintained at a temperature of (25 ± 1)°C. The duration of swimming while carrying this additional weight was carefully timed. If the mice failed to surface after a period of 5 s, the experiment was promptly terminated, and the mice were gently removed from the water to prevent drowning.

### 3.10 Experimental sleep testing of mice

For the pentobarbitone-induced sleep test, mice were given an intraperitoneal injection of pentobarbital sodium at a threshold dose (6.0 mg/mL, 10 mL/kg) to assess the sleep onset latency (SOL). Additionally, a subthreshold dose (4.5 mg/mL, 10 mL/kg) was administered to measure the total duration of sleep. These measurements served as indices of the hypnotic effect of the compound under investigation [79].

### 3.11 Detection of fatigue and sleep-related indexes

A standard protocol was followed for collecting blood samples and analyzing biochemical markers in mice. Briefly, 200 μL of blood was collected from the orbital venous plexus of the mice. To assess lactate and urea nitrogen levels in the serum, specific kits from Nanjing Jiancheng Bioengineering Research Institute Co. Ltd. (Nanjing, China) were utilized. Before tissue sampling, the mice were anesthetized through intraperitoneal injection of 1% pentobarbital sodium at a dose of 40 mg/kg. Following anesthetization, euthanasia was carried out through cervical dislocation. The livers and hind leg muscle tissues were then carefully excised, rinsed with phosphate buffer (supplied by Dalian Meilun Biotechnology Co. Ltd, Dalian, China), and patted dried with filter paper.

To evaluate the glycogen content and lipid peroxidation in the livers and muscle tissues, Glycogen Detection kits (Nanjing Jiancheng Bioengineering Research Institute Co. Ltd, China) and Lipid Peroxidation MDA Assay kits (Biyuntian Biotechnology Co. Ltd, Shanghai, China) were employed, respectively. The experiments were conducted meticulously, adhering strictly to the manufacturer's instructions to ensure accurate and reliable results.

### 3.12 Biosafety in mice

The biosafety of *Ganoderma lucidum* recipes in mice was thoroughly assessed through a series of experiments. Over a period of 14 days of continuous administration, the mice were monitored daily for any mortality. According to the protocol established by Li, N et al. [80], crucial organs such as the heart, liver, spleen, lung and kidney were carefully excised from the mice upon the completion of the experimental period. These organs were subsequently dehydrated, fixed, and embedded in paraffin to prepare histological sections. The paraffin sections were then dewaxed and stained with hematoxylin-eosin (H&E), a standard histological staining technique that enables clear visualization of cellular structures. Finally, the stained sections were meticulously examined under a microscope for a comprehensive histological analysis. This process enabled the detection of any potential abnormalities or pathological changes in the organs, providing crucial information on the safety profile of the Ganoderma lucidum recipes in mice.

### 3.13 Statistical analysis

All data were represented as group means along with the standard errors of the mean (S.E.M). To evaluate the weight-bearing swimming time, sleep onset latency, and sleep time among mice treated with GE, GB, GS, and PH, a one-way analysis of variance (ANOVA) was employed. For the remaining experiments, a two-way ANOVA followed by the Newman-Keuls multiple range test was utilized for statistical analysis. All statistical analyses were conducted using a statistics software program (GB-STAT 9.0; Dynamic Microsystems, Inc., Silver Spring, MD) to ensure the accuracy and reliability of the results.

## 4 Results

### 4.1 Effects of PGB and GBS Ganoderma lucidum recipes and their main components on weight-bearing swimming time and sleep of mice

In this study, the anti-fatigue properties of various Ganoderma recipes and their components were explored by assessing the weight-bearing swimming time of mice. Our findings reveal that a 14-day treatment with PGB significantly increased the swimming endurance of mice in a dose-dependent manner (Fig. 2A). This indicates that PGB treatment can effectively enhance exercise tolerance and thus improve the anti-fatigue capacity of mice. Contrastingly, mice treated with GBS did not exhibit a significant difference in swimming time compared to the control group, suggesting that its anti-fatigue effect was not pronounced (Fig. 2A). However, other Ganoderma components, such as Broken Ganoderma spore powder (GB), *Ganoderma lucidum* extract (GE) and Ganoderma sinensis (GS), significantly extended the weight-bearing swimming time of mice, demonstrating a significant anti-fatigue effect (Fig. 2B). Given that different *Ganoderma lucidum* extracts have exhibit anti-fatigue or sleep-aiding activities, we postulate that the various recipes containing *Ganoderma lucidum* has the potential to enhance anti-fatigue or sleep-aiding benefits.

**Fig. 1:**
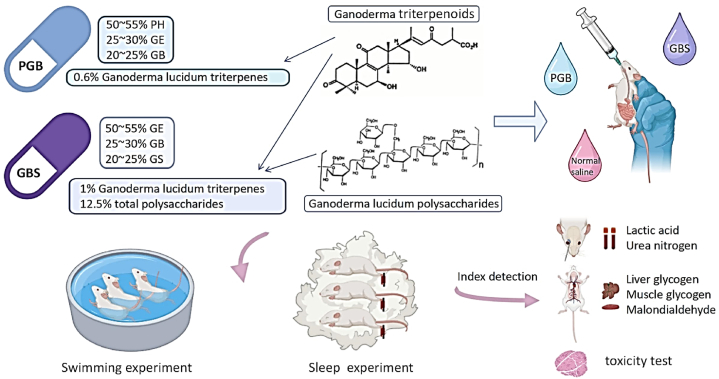
Schematic diagram of experimental plan and technical procedure.

**Fig. 2:**
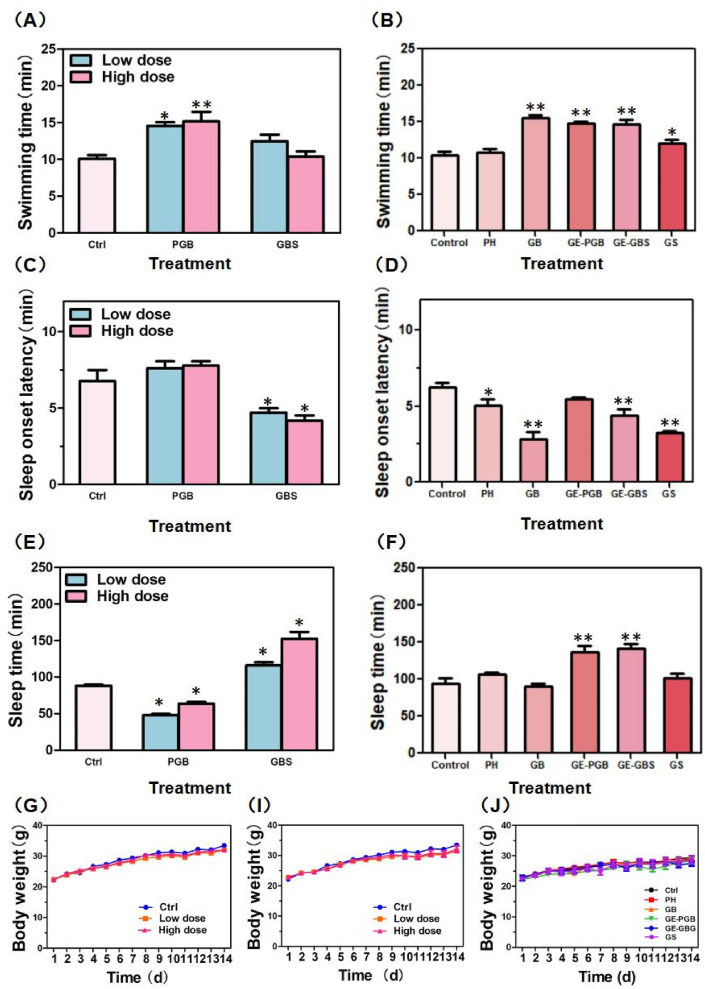
Effect of *Ganoderma lucidum* recipes PGB and GBS, along with their primary components such as GE, GB, PH and GS, on various physiological aspects of mice, including weight-bearing swimming time, sleep time (A and B), sleep onset latency (C and D), and sleep duration measurements (E and F). Effects of PGB, GBS and the main components, including PG, GB, GE and GS, on the body weight of mice (G–J). The dosages were classified as low (253 mg/kg) and high (506 mg/kg) to assess varying concentrations' effects. The concentration of PGB-L or GBS-L was set at 253 mg/kg, while PGB-H or GBG-H was administered at 506 mg/kg. The concentrations of individual components were aligned with their respective concentrations in the *Ganoderma lucidum* recipes. GE was administered at two concentrations (GE-PGB and GE-GBS) due to its varying quantities in the PGB and GBS recipes. The specific data presented in Fig. 1 (A–F) were comprehensively summarized in Table 2. * and ** indicate significant differences (at *p* < 0.05 and *p* < 0.01, respectively) from the control group.

To assess the impact of *Ganoderma lucidum* recipes and their components on sleep, direct sleep-inducing tests and pentobarbitone-induced sleep tests were conducted in mice. In these experiments, pentobarbital sodium was administered at threshold (6.0 mg/mL, 10 mL/kg) and subthreshold (4.5 mg/mL, 10 mL/kg) doses to evaluate its hypnotic properties. Mice treated with GBS for 14 days exhibited a dose-dependent significant reduction in sleep onset latency (SOL), demonstrating the recipe's effectiveness in promoting sleep (Fig. 2C). Additionally, a dose-dependent increase in the loss of righting reflex (LORR) duration was observed (Fig. 2E), indicating that GBS also prolonged sleep duration. Conversely, 14-day PGB treatment did not alter SOL, but shortened LORR duration within a specific dose range (Fig. 2C and E). Among the comparison groups, all four primary components of *Ganoderma lucidum* recipes (PH, GB, GE and GS) showed some degree of reduction in SOL, with GB exhibiting the most significant effect (Fig. 2D). Notably, only *Ganoderma lucidum* extract (GE) had a significant impact on sleep duration (Fig. 2F). These experimental results suggest that combining *Ganoderma lucidum* extract with various components result in diverse anti-fatigue or sleep effects. After 14 days of continuous administration, no significant differences were observed in the body weights of the tested mice (Fig. 2G–J). This finding suggests that neither PGB, GBS nor the main components of *Ganoderma lucidum* recipes (PH, GB, GE and GS) had a notable impact on the body weight of the mice.

### 4.2 Effects of Ganoderma lucidum recipes on lactate and urea nitrogen levels in serum

**Fig. 3:**
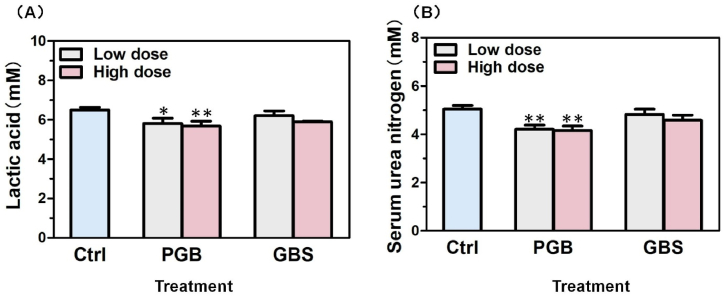
Effect of *Ganoderma lucidum* recipes (PGB or GBS) on (**A**) lactate and (**B**) urea nitrogen levels in the serum of mice. Two different concentrations of these recipes were tested: PGB-L/GBG-L at 253 mg/kg (low concentration) and PGB-H/GBG-H at 506 mg/kg (high concentration). The concentrations of the individual components were adjusted to reflect their relative proportions in the *Ganoderma lucidum* recipes. GE was tested at two concentrations (GE-PGB and GE-GBS) due to its varying quantities in the PGB and GBS recipes. The specific data were comprehensively summarized in Table 2.

In this experiment, we evaluated the serum lactate and urea nitrogen levels in mice after a 14-day administration of Ganoderma recipes (PGB or GBS). Notably, a dose-dependent reduction in serum lactate levels was observed in mice treated with PGB (Fig. 3A). This significant decrease suggests that PGB can effectively delay fatigue onset and enhance exercise endurance. In contrast, no significant changes in serum lactate levels were detected between the GBS-treated groups and the control group. Furthermore, the serum urea nitrogen levels in all PGB-treated groups exhibited a significant decrease, whereas no significant difference was observed between the GBS-treated group and the control group (Fig. 3B). These findings further support the anti-fatigue effects of PGB on mice.

### 4.3 Effects of Ganoderma lucidum recipes on glycogen and MDA in the livers and muscle tissues in mice

**Fig. 4:**
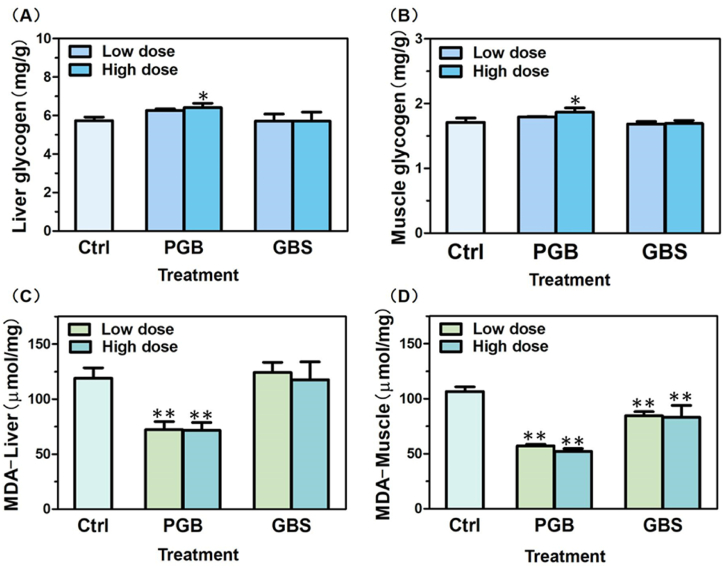
Effects of PGB and GBS on glycogen and malondialdehyde levels in the livers and muscle tissues of mice. The levels of hepatic glycogen (**A**), muscle glycogen (**B**), malondialdehyde in the livers (**C**) and muscle tissues (**D**) were examined. The concentrations of PGB-L/GBG-L and PGB-H/GBG-H were set at 253 mg/kg and 506 mg/kg, respectively. The concentration of each component aligned with its proportion in the *Ganoderma lucidum* recipes' dose concentrations. GE was evaluated at two concentrations (GE-PGB and GE-GBS) to account for its varying contents in the PGB and GBS recipes. The comprehensive data obtained from this study are summarized in Table 2.

In this study, we examined the impact of Ganoderma recipes (PGB or GBS) on hepatic glycogen and muscle glycogen reserves in mice by assessing their respective glycogen contents after a 14-day administration period. Notably, a significant increase in both hepatic glycogen (Fig. 4A) and muscle glycogen (Fig. 4B) levels in mice treated with PGB at the high dose. Conversely, no notable changes were detected in either hepatic or muscle glycogen content in mice treated with GBS compared to the control group.

Subsequently, we measured the content of MDA in the livers and muscle tissues of mice treated with PGB or GBS. The findings revealed a significant reduction in MDA levels in both livers and muscle tissues following PGB administration (Fig. 4C-D). However, GBS administration only significantly decreased MDA content in the muscle tissues of mice. These results indicate that PGB and GBS possess varying degrees of oxygen-free radical scavenging capabilities in the livers and muscles, exhibiting differential effects in reducing oxidative stress damage in tissues.

### 4.4 Biosafety of Ganoderma lucidum recipes in mice

**Fig. 5:**
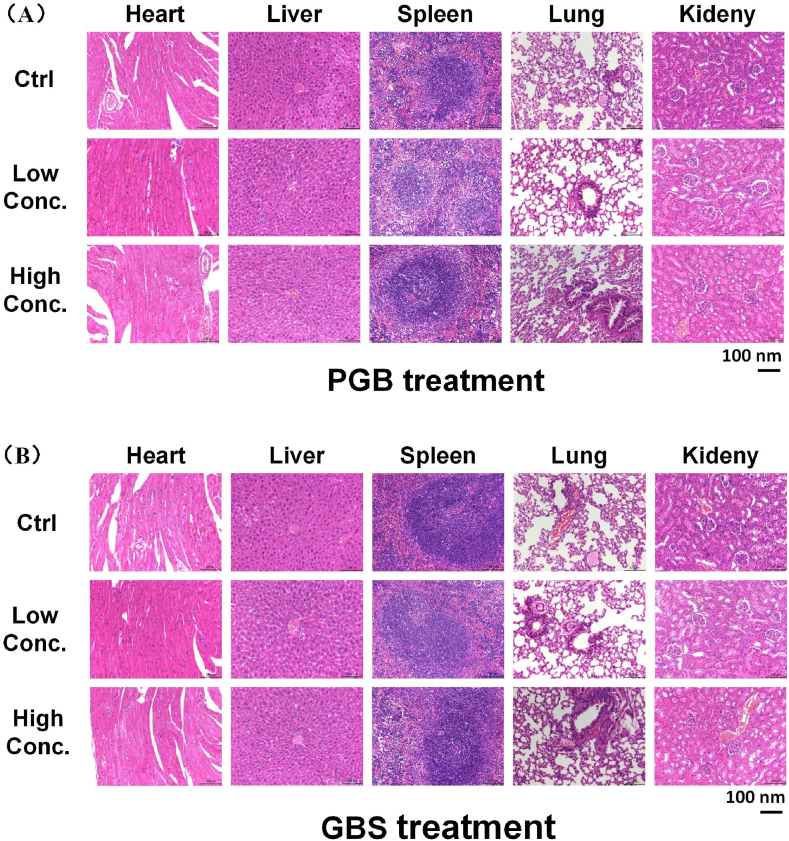
Biosafety of PGB and GBS in mice. Representative H&E staining images of vital organs (including hearts, livers, spleens, lungs and kidneys) in mice treated with PGB (**A**) or GBS (**B**) for 14 days (with a 200-times magnification).

The biosafety of PGB and GBS was meticulously assessed by monitoring the mortality rates of mice over a continuous 14-day administration period. After the experiment, histological analysis was conducted on various mouse tissues. including hearts, livers, spleens, lungs and kidneys using H&E staining (Fig. 5). The findings from our study reveal that, within the prescribed dose range, both PGB and GBS did not cause any notable organ damage in mice. This observation underscores the excellent biosafety profile of these formulations in this animal model.

## 5 Discussion

Exercise-induced fatigue is a prevalent physiological occurrence, typically characterized by the body's inability to sustain a particular level of functionality or exercise intensity [81]. This fatigue is often paralleled by alterations in the levels of vital energy substances, metabolites, hormones, and enzymes responsible for regulating cell metabolism and the antioxidant system. Essentially, these changes reflect a disruption in the homeostasis of the body's internal environment, leading to physical discomfort [82]. Prolonged or severe fatigue can cause endocrine imbalances and compromise immune function, resulting in organic diseases, which significantly impact people's physical well-being and their daily lives.

Sleep, being a fundamental biological behavior for maintaining vital life activities, plays a crucial role in restoring physical condition and overcoming fatigue [83]. Nevertheless, according to “White Paper on Exercise and Sleep in 2021” published by the Chinese Sleep Research Society [84], over 300 million individuals in China suffer from insomnia, primarily exhibiting symptoms such as difficulty initiating and maintaining deep sleep. Therefore, enhancing sleep quality and alleviating the stress caused by fatigue has become a pressing concern for individuals in today's society.

*Ganoderma lucidum*, a healthcare fungus with a rich historical background, holds significant importance in various aspects of health maintenance. Its role is paramount in the prevention and treatment of cardiovascular diseases, neurasthenia, anti-tumor therapy, liver protection and more. This fungus boasts a complex array of chemical components, endowing it with diverse pharmacological effects and a wealth of active substances and nutrients. Among these, polysaccharides and triterpenoids stand out as the two principal active components. *Ganoderma lucidum* polysaccharide exhibits remarkable abilities in scavenging oxidative free radicals, suppressing lipid peroxidation [85], bolstering immune effector cells [85,86] and enhancing overall immune function, which contributes to anti-fatigue effects [87]. On the other hand, *Ganoderma lucidum* triterpenes protects nerve cells from harm by inhibiting the production of excitatory neurotransmitters [88] and pro-inflammatory mediators [85], while exerting sedation, sleep-aiding, anti-inflammatory, and neuroprotective effects [89,90]. To further unlock the medicinal and health-promoting potential of *Ganoderma lucidum*, we evaluated two of its recipes. We aim to establish a theoretical and experimental foundation for the development of functional *Ganoderma lucidum* recipes that specifically target fatigue reduction and sleep enhancement.

In the preliminary experiment, we initially attempted a higher dose of PGB and GBS (759 mg/kg) in treating the experimental mice. However, upon analyzing the H&E staining results of the mice tissues, we observed cavities in the renal tissue, indicating toxicity to the kidney. As a result, the dosages were lowered to 253 and 506 mg/kg, respectively, for subsequent treatment. Our observations revealed that PGB treatment effectively augmented the stores of energy substances, specifically hepatic glycogen and muscle glycogen while reducing the accumulation of lactate and urea nitrogen. This enhancement led to improved exercise endurance and antioxidant capacity, demonstrating its anti-fatigue effects. These mechanisms are aligned with previous reports in the literature [91,92]. Additionally, PGB treatment notably shortened the duration of loss of righting reflex (LORR) in mice, potentially linked to its anti-fatigue properties.

In contrast, GBS treatment significantly shortened the sleep onset latency (SOL) and prolonged the LORR duration in mice, indicating its sedative and hypnotic effects. Notably, we observed a decrease in the level of MDA in the livers of mice treated with GBS. Prior studies by S. Rajendiran and colleagues have reported a significant increase in MDA levels in patients experiencing sleep deprivation [37]. Our findings, where the decrease in MDA levels coincided with prolonged sleep time in the GBS-treated mice group, are in line with their observations, suggesting that the MDA level in muscles may serve as a crucial indicator for assessing sleep quality.

Based on Table 2, treatment with high-dose PGB resulted in notable improvements in several key biochemical markers. Specifically, serum lactate and serum urea nitrogen levels were reduced by 14.4% and 17.4%, respectively. Additionally, hepatic glycogen and muscle glycogen were increased by 11.8% and 9.2%, while MDA levels in the livers and muscles decreased by 39.9% and 51.1%, respectively. On the other hand, high-dosage GBS treatment solely reduced MDA levels in the livers by 22.1%, without significant changes in other indicators. Comparing these findings with recently reported literature, we observe that the reduction rates of serum lactate and serum urea nitrogen in our study fall within safe ranges, below 34.5% and 25.2%, respectively [[93], [94], [95]]. Similarly, the increases in hepatic glycogen and muscle glycogen were within acceptable limits, below 51.0% and within 45.0%, respectively [93,94,96]. Furthermore, the reduction rates of MDA levels in the livers and muscles in our study were also within safe ranges, below 52.8% and 54.7%, respectively [55,97,98]. Based on these results, we conclude that the changes in these biochemical markers in the PGB or GBS-treated mice of this study are within safe ranges. Additionally, the H&E staining images of various mouse tissues indicated no obvious adverse effects from PGB or 10.13039/100019980GBS, further supporting the good biological safety of these two *Ganoderma lucidum* formulae.

**Table 2:** Anti-fatigue and sleep-aiding effects of PGB and GBS, as well as the associated index levels in mice.

		Control	PGB-L	PGB-H	GBS-L	GBS-H
Swimming time (min)		10.10 ± 0.45	14.56 ± 0.42*	15.18 ± 1.18**	12.59 ± 0.69	10.38 ± 0.62
Sleep time (min)	Sleep latency Duration time	6.77 ± 0.65 88.26 ± 1.27	7.61 ± 0.40 48.71 ± 1.63*	7.78 ± 0.25 64.96 ± 2.22*	4.70 ± 0.26* 116.08 ± 3.66*	4.17 ± 0.32* 152.50 ± 8.30*
Serum lactate (mM)		6.50 ± 0.13	5.82 ± 0.24*	5.64 ± 0.24*	6.21 ± 0.22	5.89 ± 0.03
Urea nitrogen (mM)		5.05 ± 0.14	4.28 ± 0.16**	4.17 ± 0.16**	4.82 ± 0.20	4.58 ± 0.19
Hepatic glycogen (mg/g)		5.77 ± 0.18	6.28 ± 0.07	6.41 ± 0.21*	5.72 ± 0.32	5.72 ± 0.40
Muscle glycogen (mg/g)		1.71 ± 0.06	1.79 ± 0.01	1.87 ± 0.06*	1.69 ± 0.03	1.69 ± 0.04
Liver-MDA (μM)		118.98 ± 8.65	72.21 ± 6.34**	72.05 ± 7.11**	124.25 ± 7.47	122.67 ± 15.36
Muscle-MDA (μM)		105.80 ± 4.09	57.07 ± 1.38**	52.15 ± 2.21**	81.82 ± 2.41**	83.01 ± 9.38**

Note: These recipes were tested at two different concentrations: PGB-L and GBS-L at 253 mg/kg, PGB-H and GBS-H at 506 mg/kg * and ** indicate significant differences at *p* < 0.05 and *p* < 0.01, respectively, as compared with control group.

In our study, we delved into the anti-fatigue and sleep-aiding mechanisms of PGB and GBS, revealing the diverse effects of different Ganoderma lucidum formulae. This provides valuable experimental evidence for the clinical compatibility of *Ganoderma lucidum* with traditional Chinese medicine. At the same time, we are also aware of the limitations of our research. For instance, further exploration is needed to fully understand the complex interactions between the formulae and the body's metabolic processes, as well as to clarify their long-term effects and safety profiles. To overcome these limitations and maximize the effectiveness of *Ganoderma lucidum*, we plan to explore the use of nano-to-micro biomaterials in future research to protect the formula and reduce losses in the gastrointestinal tract. This will enhance the bioavailability of active components and potentially improve therapeutic outcomes.

## 6 Conclusion

In summary, our findings reveal that PGB, enriched with Ganoderma polysaccharides, exhibits notable anti-fatigue activity. Conversely, GBS, formulated with extracts from GS and *Ganoderma lucidum*, demonstrates a pronounced sleep-improving effect. These results indicate that *Ganoderma lucidum* based recipes can effectively target either fatigue or sleep issues, depending on the specific combination of compatible ingredients. As such, they present promising applications as health-promoting formulations.

## Ethics statement

All procedures were conducted by the Guidelines for Care and Use of Laboratory Animals approved by the Animal Ethics Committee in Fujian University of Traditional Chinese Medicine (2022-16).

## Data availability statement

The data of this study is available from the corresponding authors upon reasonable request.

## CRediT authorship contribution statement

**Kexin Li:** Writing – original draft, Software, Investigation, Formal analysis, Data curation. **Wenzhen Liu:** Writing – original draft, Investigation, Data curation. **Changhui Wu:** Software, Conceptualization. **Le Wang:** Methodology. **Yunmei Huang:** Methodology. **Ye Li:** Software. **Huimin Zheng:** Investigation, Conceptualization. **Yanyu Shang:** Investigation. **Lei Zhang:** Writing – review & editing, Funding acquisition. **Zhuo Chen:** Writing – review & editing, Project administration, Funding acquisition.

## Declaration of competing interest

The authors declare that they have no known competing financial interests or personal relationships that could have appeared to influence the work reported in this paper.
